# A new generation of recombinant polypeptides combines multiple protein domains for effective antimicrobial activity

**DOI:** 10.1186/s12934-020-01380-7

**Published:** 2020-06-05

**Authors:** Ramon Roca-Pinilla, Adrià López-Cano, Cristina Saubi, Elena Garcia-Fruitós, Anna Arís

**Affiliations:** Department of Ruminant Production, Institute of Agriculture and Food Research (IRTA), 08140 Caldes de Montbui, Spain

**Keywords:** Antimicrobial peptides, Antimicrobial resistance, Inclusion bodies, Multidomain protein, Solubilization, Recombinant production, Protein nanoclusters

## Abstract

**Background:**

Although most of antimicrobial peptides (AMPs), being relatively short, are produced by chemical synthesis, several AMPs have been produced using recombinant technology. However, AMPs could be cytotoxic to the producer cell, and if small they can be easily degraded. The objective of this study was to produce a multidomain antimicrobial protein based on recombinant protein nanoclusters to increase the yield, stability and effectivity.

**Results:**

A single antimicrobial polypeptide JAMF1 that combines three functional domains based on human α-defensin-5, human XII-A secreted phospholipase A2 (sPLA_2_), and a gelsolin-based bacterial-binding domain along with two aggregation-seeding domains based on leucine zippers was successfully produced with no toxic effects for the producer cell and mainly in a nanocluster structure. Both, the nanocluster and solubilized format of the protein showed a clear antimicrobial effect against a broad spectrum of Gram-negative and Gram-positive bacteria, including multi-resistant strains, with an optimal concentration between 1 and 10 µM.

**Conclusions:**

Our findings demonstrated that multidomain antimicrobial proteins forming nanoclusters can be efficiently produced in recombinant bacteria, being a novel and valuable strategy to create a versatile, highly stable and easily editable multidomain constructs with a broad-spectrum antimicrobial activity in both soluble and nanostructured format.

## Background

The growing number of antibiotic-resistant pathogens is a pressing healthcare challenge. As a consequence, the development of novel antimicrobial drugs is more necessary than ever, especially against multidrug-resistant (MDR) microorganisms. One source of potential broad-spectrum antibacterials with increasing promise are antimicrobial peptides (AMPs), which are peptides from the innate immune system of nearly all multicellular organisms [1, 2]. AMPs also known as host defense peptides (HDPs), are cationic amphiphilic peptides [3]. These positively charged peptides are classified into defensins (alpha-defensins and beta-defensins), cathelicidins, and histatins [1]. Because they interact and disrupt the negatively charged bacterial cell envelope, they have broad-spectrum antibacterial activity [4, 5]. Although AMPs hold therapeutic potential, even against MDR bacteria [4–6], important drawbacks largely hinder final in vivo applications [3]. Most of the currently used AMP are produced by chemical synthesis, being molecules highly susceptibility to proteolytic degradation by microbial and host enzymes (short half-life) [7]. Besides, due to the high doses needed they are frequently toxic and high production costs are still a problem for large-scale development [8]. Alternatively, AMPs can be recombinantly produced [9–11]. Still, their small size makes them easily degradable, and their recombinant production is limited because they are toxic for the producer bacterial cell due to their antimicrobial nature. To address these shortcomings, different approaches to make the production of recombinant AMPs linked to a carrier that stabilizes the peptide have been described. Some examples of carriers for these fusion proteins are small ubiquitin-like modifier (SUMO), thioredoxin (Trx), glutathione *S*-transferase (GST), biotin carboxyl carrier protein (BCCP), green fluorescent protein (GFP), calmodulin and human serum albumin [12–15]. Another strategy to efficiently produce these peptides is the use of acidic partners [16]. In all the cases, these carriers or partners help to overcome the toxicity of the AMP and at the same time increase their protein expression yields [16–18]. However, to retrieve the AMP of interest, it is often necessary to remove these carriers, which requires expensive enzymatic cleavage or toxic reagents [19].

To address this gap, here, we have explored a new strategy for the production of a recombinant antimicrobial protein based on a multidomain polypeptide that combines different functional domains in a single molecule but without a carrier protein. The combination of several domains has been previously reported for other proteins [20–23], but none of them for antimicrobial treatment purposes. Additionally, taking into consideration the specific requirements of price, stability, toxicity, effectiveness, and delivery that appear to be key parameters in the development of a new generation of antimicrobials [3], we have added aggregation-seeding domains based on leucine zippers that increase the recombinant production of the antimicrobial molecules as protein nanoclusters (also known as inclusion bodies (IBs)) [24]. IBs are non-enveloped, porous and mechanically stable protein nanoparticles, mainly formed by the polypeptide of interest and generated during recombinant protein production process, having the potential to be a protein-slow release form when administered [25]. Another advantage of this protein format is its production through a one step-process. This means that, in contrast to most of encapsulation strategies, which involve two separate processes (the production of the carriers and the biomolecule separately), IB production is achieved in one single step. Finally, it has also been previously proven that IBs can be used as a source of soluble protein after a mild extraction protocol in non-denaturing conditions [26, 27].

## Results and discussion

### Construct design and protein production of JAMF1 as protein nanocluster

**Fig. 1:**
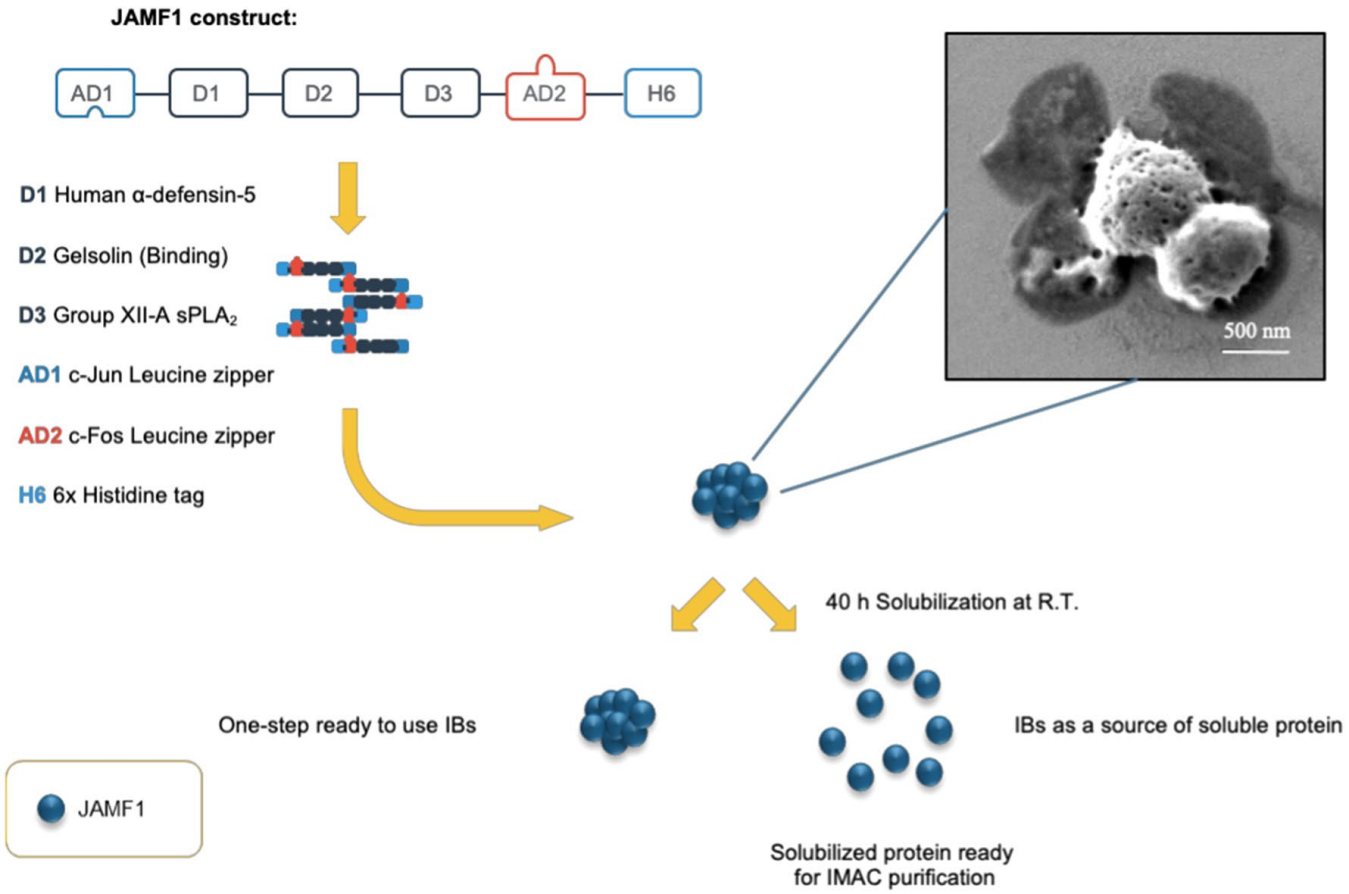
Construct design. Schematic representation of JAMF1 protein construct and protein production format. Inset image: FESEM micrography of purified JAMF1 nanoparticles

Our construct, named JAMF1 and formed by the combination of an HDP (HD5) [28], a bacterial binding domain (gelsolin) [29] and an enzymatic antimicrobial peptide (sPLA_2_) [30], flanked by two aggregation-seeding domains (c-Jun and c-Fos leucine zippers at N- and C-terminal, respectively), has been designed (Fig. 1 and Additional file 1: Figure S1). During their production in a recombinant bacterial system, no toxicity effects were observed in the producer cell (data not shown) and, as desired, the multidomain JAMF1 protein (54 kDa) was mainly produced as protein IBs (Additional file 1: Figure S2). The percentage of JAMF1 aggregation as IBs was 74 ± 3.1% of the total multidomain protein overproduced, reaching a yield of 96.5 mg/l and a purity of 95% once purified. FESEM micrographs of the purified JAMF1 IBs showed a porous morphology with a round shape and a particle size of around 500 nm (Fig. 1).

### Antibacterial activity of JAMF1 nanoclusters

**Fig. 2:**
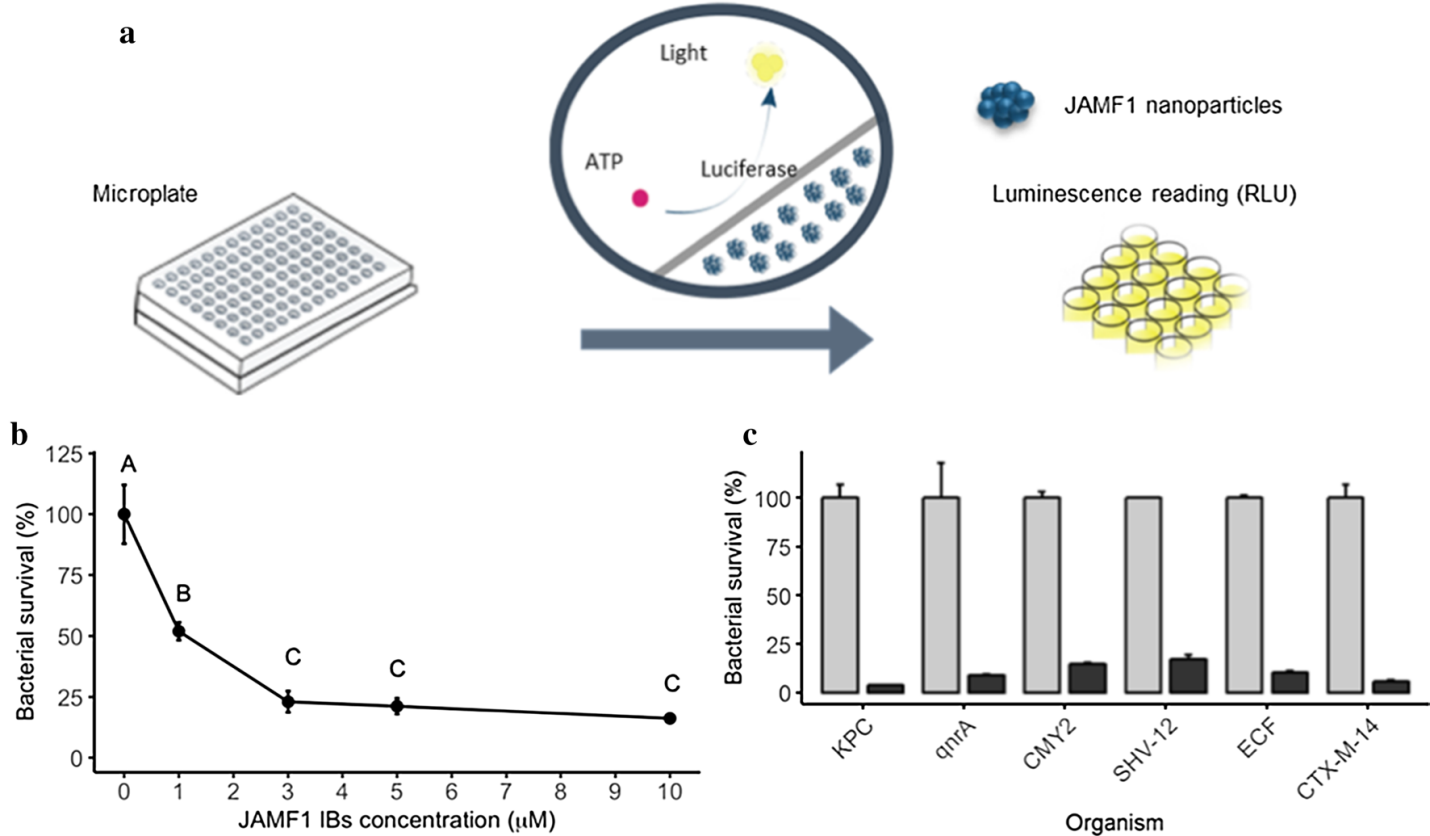
Antibacterial activity of JAMF1 nanoclusters. **a** Graphic representation of the BacTiter-Glo™ Microbial Cell viability assay. **b** Bacterial survival (%) of *E. coli* DH5α in the presence of JAMF1 IBs at a range of 0-10 µM. Different letters describe significant differences (p ≤ 0.01). **c** Bacterial survival of KPC, qnrA, CMY2, SHV-12, ECF and CTX-M-14 bacterial strains in the presence of 10 µM of JAMF1 IBs. Survival of JAMF1 treated bacterial cells (black bars) is significantly different from the negative control (grey bars) (p ≤ 0.001)

To determine the antimicrobial activity of JAMF1 IBs, we evaluated the survival of both Gram-positive and Gram-negative pathogenic bacteria treated with JAMF1 IBs, following the procedure described in Fig. 2a. First, the survival of an *E. coli* DH5α model strain in presence of increasing concentrations of JAMF1 IBs was determined and a dose dependent effect was observed (p ≤ 0.01) (Fig. 2b). Using the concentration of JAMF1 IBs giving the lowest values of *E. coli* survival (10 μM), we tested the antibacterial effect of these nanoparticles with different Gram-positive strains, including extended-spectrum beta-lactam-resistant *Enterococcus* spp. (SHV-12), extended-spectrum beta-lactam-resistant *Enterococcus* spp. (CTX-M-14), and *E. faecalis* (ECF), and Gram-negative strains, including Carbapenem-resistant *Klebsiella pneumoniae* (KPC), quinolone-resistant *K. pneumoniae* (qnrA), and extended-spectrum beta-lactam-resistant *E. coli* (CMY2) (Fig. 2c). In all strains tested we observed a clear decrease in the survival (p ≤ 0.001), reaching viability reduction values of 96.3 ± 0.2% for KPC, 91 ± 0.2% for qnrA, 85.3 ± 0.6 for CMY2, 82.8 ± 2% for SHV-12, 89.8 ± 0.9% for ECF, and 94.4 ± 0.7% for CTX-M-14 (Fig. 2c).

### Anti-biofilm activity of JAMF1 nanoclusters

**Fig. 3:**
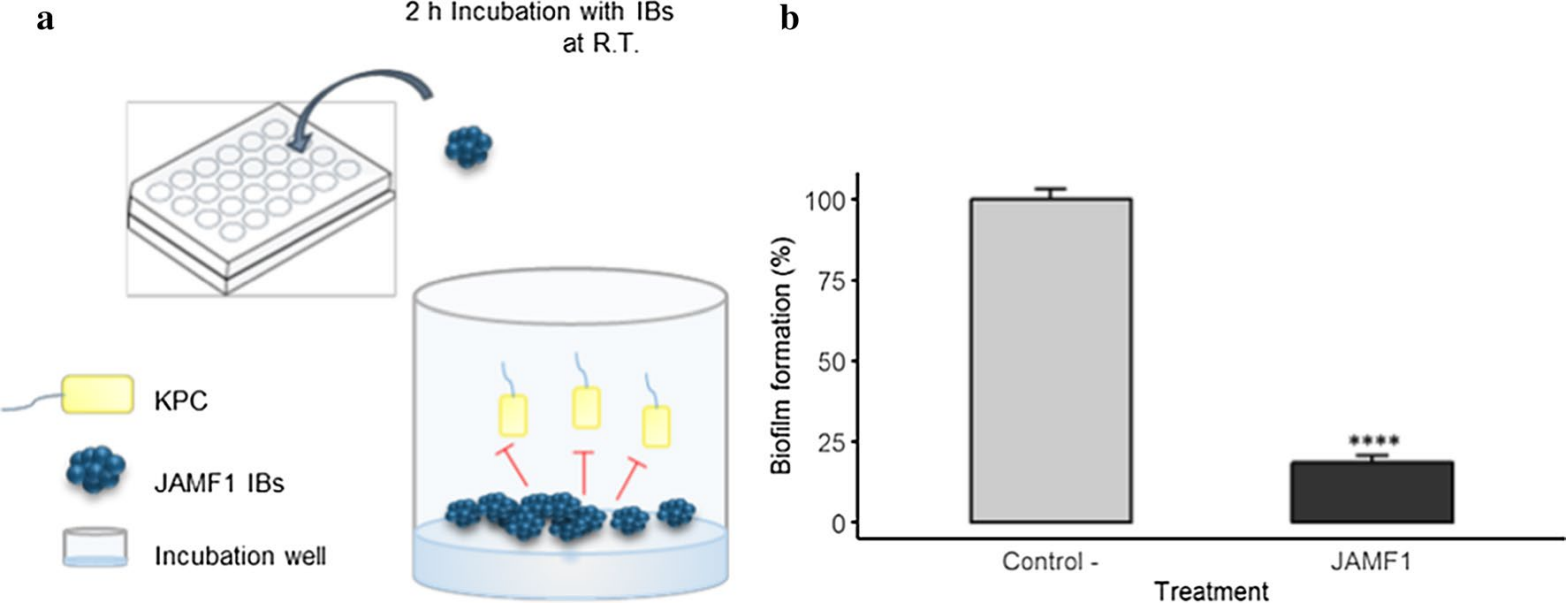
Anti-biofilm activity of JAMF1 nanoclusters. **a** Biofilm inhibition assay. Plate wells were incubated for 2 h with JAMF1 IBs and then a diluted (1:200) KPC cell culture with 0.2% glucose was added and incubated for 24 h to allow biofilm formation. **b** Biofilm formation ability (%) of KPC after treating plastic wells with JAMF1 IBs (black bar) vs non-treated wells (grey bar). ****Indicates significant differences (p ≤ 0.0001)

To further evaluate the potential of this new class of antimicrobial proteins we have assessed the capacity of JAMF1 nanoclusters to inhibit biofilm formation. For that, KPC was grown in multiwell plates in which JAMF1 IBs were previously immobilized, as detailed in Fig. 3a. The results obtained showed a decrease of 81.4 ± 2.3% in biofilm formation (p ≤ 0.0001) when surfaces were decorated with JAMF1 IBs (Fig. 3b), which confirms that antimicrobial nanoclusters are also active when deposited on plastic surfaces to inhibit biofilm formation.

Several works have studied the IBs appealing features in contexts such as cancer [31], tissue regeneration [32], and immunostimulation [33], demonstrating its great potential as a new biomaterial. However, to the best of our knowledge, this is the first study exploring the antimicrobial effect of a multidomain protein embedded in IBs. Whereas previous studies have used fusion partners such as SUMO [12], Trx, GST [13], and human serum albumin [14] to overcome the difficulties to express relatively short peptides, the current work shows that it is also possible to produce a non-toxic and stable AMP-based molecule as a combination of several AMPs. This offers versatility in the construction of molecules and the possibility to explore several combinations to merge complementary antimicrobial activities without the need to use biologically irrelevant carrier proteins. The production of this new generation of antimicrobial multidomain polypeptides as nanoclusters seems to be a good strategy to escape proteolytic and host-toxicity pathways in the recombinant bacterial host.

### Solubilized JAMF1 antibacterial activities

**Fig. 4:**
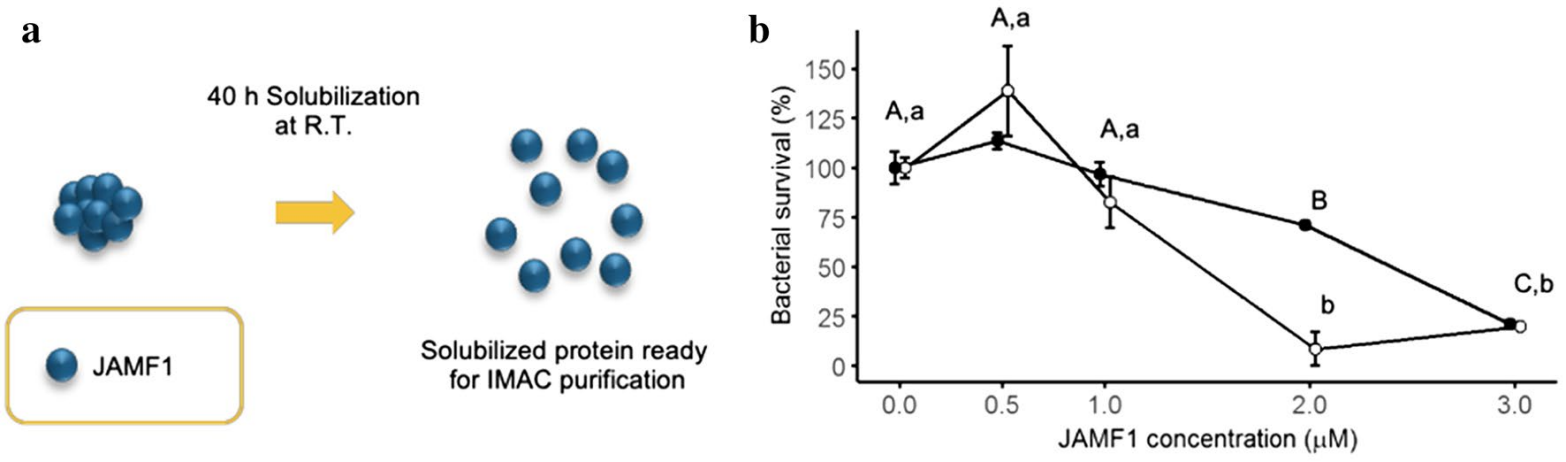
Solubilized JAMF1 antibacterial activities. **a** Schematic representation of JAMF1 IB solubilization at RT. **b** Bacterial survival (%) of *E. coli* DH5α and KPC at different concentrations (0, 0.5, 1, 2 and 3 µM) of solubilized JAMF1. Capital letters depict significant differences for *E. coli* DH5α (p ≤ 0.0001) and lower case for KPC (p ≤ 0.001). Filled circles correspond to *E. coli* DH5α and empty circles correspond to KPC

For some specific applications a soluble version of the multidomain antimicrobial polypeptide would be more appropriate than the nanostructured or IBs version, for example those applications in which an intravenous administration of the protein would be necessary. Interestingly, the IB format has also been previously described as a reservoir of functional protein [25] and, in consequence, an appealing source of the soluble version of proteins tricky to produce, available after using a mild-extraction protocol [26]. Considering that JAMF1 is mainly produced as IBs (74% of the total protein overproduced), we also explored if JAMF1 IBs could be used as a source to obtain pure antimicrobial multidomain polypeptide in its soluble form using a non-denaturing protocol and evaluate its antimicrobial potential (Fig. 4a). The results proved that the purified soluble version could not only be isolated from IBs (Additional file 1: Figure S2) but also showed antimicrobial activity against either *E. coli* (p ≤ 0.0001) and KPC (p ≤ 0.001) in a dose-dependent manner, where the growth inhibition reached values of 78.7 ± 2% and 91.3 ± 8.5% for *E. coli* and KPC at 3 and 2 μM, respectively (p < 0.0001) (Fig. 4b). Interestingly, the antimicrobial efficiency of this new multidomain antimicrobial molecule is pretty similar at 3 μM, achieving efficiencies of around 78% in both cases (Figs. 2b and 4b). This contrasts with the majority of examples comparing the activity of soluble and IB formats, in which the soluble version is usually more biologically active that its nanoclustered counterpart [34].

## Conclusions

Our findings demonstrated that multidomain antimicrobial proteins produced as nanoclusters can be efficiently obtained in recombinant bacteria, being a valuable strategy to create a versatile, highly stable and easily editable constructs with antimicrobial activity against both Gram-positive and Gram-negative bacteria. Moreover, we have proven that the antimicrobial protein forming the IBs can be easily solubilized to obtain also active proteins in its soluble form.

## Methods

### Bacterial strains and medium

*Escherichia coli* BL21 (DE3) was used for heterologous protein expression. Strains used for antibacterial and antibiofilm activity assays were *E. coli* DH5α, Carbapenem-resistant *Klebsiella pneumoniae* (KPC), quinolone-resistant *K. pneumoniae* (qnrA), extended-spectrum beta-lactam-resistant *E. coli* (CMY2), extended-spectrum beta-lactam-resistant *Enterococcus* spp. (SHV-12), extended-spectrum beta-lactam-resistant *Enterococcus* spp. (CTX-M-14) and *E. faecalis* (ECF). All strains were grown in Brain–Heart Infusion (BHI) broth (Scharlau, Barcelona, Spain), except for *E. coli* strains, which were grown in Luria–Bertani (LB) medium.

### Genetic construct design

From N-terminal to C-terminal, the gene for the JAMF1 (Additional files 2, 3, 4) construct consisted of the sequences encoding Jun257-318 (Uniprot entry P05412), human α-defensin-5 (HD5) precursor (Uniprot entry Q01523), gelsolin188-196 (Uniprot entry P06396), human XII-A secreted phospholipase A_2_ (sPLA_2_) precursor (Uniprot entry Q9BZM1) and Fos118-210 (Uniprot entry P01100). A linker sequence (SGGGSGGS) was used between each of the domains and a C-terminal H6-Tag for protein purification. The fusion construct was codon optimized by GeneArt (Lifetechnologies, Regensburg, Germany) and cloned into pET22b (Amp^R^) (Novagene, Darmstadt, Germany) vector.

### Inclusion body production and purification

*Escherichia coli* BL21 (DE3)/pET22b-JAMF1 (Additional file 3) culture (0.5 l) was grown at 37 °C and 250 rpm in LB broth with ampicillin at 100 μg/mL. Protein expression was induced by 1 mM isopropyl-β-d-thiogalactoside (IPTG) at an OD_600_ = 0.4–0.6. Cultures were grown 3 h post-induction and after that processed as previously described [35]. Briefly, protease inhibitors (Complete EDTA-free, Roche), and phenylmethanesulphonyl-fluoride (PMSF) and lysozyme were added to the culture at a final concentration of 0.4 mM (Sigma-Aldrich) and 1 µg/ml (Sigma-Aldrich), respectively. After 2 h of incubation at 37 °C and 250 rpm the culture was centrifuged and resuspended in 50 ml of PBS supplemented with protease inhibitors. Then, the mixture was ice-jacketed and sonicated for 4 cycles of 1.5 min at 10% amplitude under 0.5 s cycles. After sonication, the mixture was frozen overnight (ON) at − 80 °C. The mixture was thawed and Triton X-100 was added (0.4% (v/v)) and incubated for 1 h at room temperature (RT). After this treatment, the mixture was frozen at − 80 °C for 2 h and then thawed between for several cycles until no viable bacterial growth was detected. After that, 125 µl of Nonidet P40 (NP-40) was added and incubated for 1 h at 4 °C. Then, DNA was removed with DNAse at a final concentration of 0.6 µg/ml and MgSO_4_ 0.6 mM for 1 h at 37 °C and 250 rpm. Samples were centrifuged at 15,000×*g* for 15 min at 4 °C. The pellet containing IBs was washed with 25 ml lysis buffer (50 mM Tris–HCl pH 8, 100 mM NaCl, 1 mM EDTA and Triton X-100 0.5% (v/v)). Finally, a centrifugation at 15,000×*g* and 4 °C for 15 min was carried out obtaining pellets that were stored at − 80 °C until analysis. Purified IBs were quantified by western blot using a monoclonal anti-His antibody (His-probe, Santa Cruz).

### IB solubilization and purification of the solubilized JAMF1

*Escherichia coli* BL21 (DE3)/pET22b-JAMF1 culture (2 l) was grown as previously described. The whole volume was centrifuged at 6000×*g* and the pellet was resuspended in 120 ml of PBS 1x in presence of protease inhibitors. Samples of 30 ml were subjected to 4 rounds of sonication for 5 min at 10% amplitude under 0.5 s cycle, intercalated by a minimum of 5 min repose in ice. Protein pellets were recovered and washed twice with distilled water. Pellets were weighted and solubilized in 0.2% *N*-lauroyl sarcosine, 40 mM Tris and protease inhibitors at a ratio of 40 ml/g of wet pellet as previously described [27]. The mixture was incubated 40 h ON at RT under agitation and the supernatant was recovered through centrifugation at 15,000×*g* for 45 min at 4 °C for further purification. NaCl and imidazole were added to the solubilized protein to equilibrate the samples with the binding buffer composition, and Immobilized Metal Affinity Chromatography (IMAC) purification was carried in an ÄKTA purifier FPLC (GE Healthcare, Chicago, IL, USA) using 1 ml HisTrap HP columns (GE Healthcare). Both the binding and the elution buffer contained 0.2% *N*-lauroyl sarcosine, and the final imidazole concentration in the elution buffer was 0.5 M. The buffer of the selected fractions was changed to 10 mM KPi (K/PO_4_ buffer pH 7.4) using a desalting column (GE Healthcare). The amount of purified protein was determined by Bradford’s assay, and the integrity of the protein analyzed by SDS-PAGE [26].

### Antibacterial activity assay

The bacterial cell viability was assessed with the BacTiter-Glo™ Microbial Cell Viability assay (Promega, Mannheim, Germany), according to the manufacturer’s protocol. Shortly, bacterial cells were grown O/N at 37 °C and 250 rpm and then diluted 1:100 in KPi buffer. Then, 150 µl from the KPi diluted cells were centrifuged in 1 ml tubes at 6200×*g* at 4 °C for 15 min. Subsequently, the supernatant was removed, and the pelleted cells were then resuspended with 150 µl of either KPi buffer (negative control) or 150 µl of JAMF1 IBs at 1, 3, 5 and 10 µM. After 5 h incubation at 37 °C in a 96-well plate, 100 µl were taken and mixed with 100 µl of the BacTiter-Glo^TM^ reagent. Finally, luminescence was measured in a microplate luminometer (LUMIstar^®^, BMG LABTECH. Ortenberg, Germany). The measured arbitrary luminescence values were normalized against the control (KPi treatment).

### Biofilm formation assay

KPC was used as a model strain. Briefly, an O/N was grown at 37 °C and 250 rpm. Before adding bacteria to a 24-well sterile plate for biofilm formation, IB-treated wells were incubated 2 h at RT with 80 µl/well of 500 µM of JAMF1 IBs. After that, bacteria from the O/N culture were diluted 1:200 in BHI supplemented with 0.2% (w/v) glucose and grown in 24-well sterile plate (400 µl final volume) and incubated at 37 °C for 24 h. After the incubation, the supernatant was removed and wells were washed three times with 500 µl NaCl 0.9%, then fixated with 500 µl methanol for 10 min at RT. Methanol was removed and the plate was dried at 37 °C for 15 min. Finally, the remaining cells in the well were stained with 1% (v/v) crystal violet for 15 min at RT, washed three times with sterile MQ-H_2_O. Finally, stained cells were diluted in 33% (v/v) acetic acid and the absorbance was measured at 595 nm [36]. All measurements were done by triplicate and in sterile conditions.

### Electron microscopy

Microdrops of JAMF1 IB suspensions were air-dried on silicon wafers and micrographed in a FESEM Zeiss Merlin (Zeiss) running at 1 kV

### Statistical analysis

For all assays, each condition was performed in triplicate and represented as the mean ± standard error of the mean. All data were checked for normality. All *p*-values correspond to ANOVA analyses, except for the biofilm formation assay where a *t*-test was performed. Letters correspond to Tukey test analyses.

## Supplementary information

**Additional file 1: Figure S1.** Aminoacidic sequence of JAMF1 for each of the construct domains. **Figure S2.** Western Blots of JAMF1. (a) Expression time course of JAMF1 at 0, 1, 3 and 5 h post-induction with IPTG in the insoluble and soluble fractions. MWM = Molecular Weight Marker (kDa), IF = Insoluble fraction, SF = Soluble fraction. (b) Western blot of the purified JAMF1 by IMAC (PP) and the purified IBs (PIBs).

**Additional file 2.** DNA coding sequence of JAMF1.

**Additional file 3.** DNA coding sequence of pET22b-JAMF1.

**Additional file 4.** JAMF1 aminoacid sequence.

## Data Availability

The datasets used and/or analysed during the current study are available from the corresponding author on reasonable request.

## Abbreviations

AMP: Antimicrobial peptide
sPLA_2_: Human XII-A secreted phospholipase A2
MDR: Multidrug resistant
IB: Inclusion body
KPC: Carbapenem-resistant *Klebsiella pneumoniae*
qnrA: Quinolone-resistant *K. pneumoniae*
CMY2: Extended-spectrum beta-lactam-resistant *Escherichia coli*
SHV-12: Extended-spectrum beta-lactam-resistant *Enterococcus* spp
CTX-M-1: Extended-spectrum beta-lactam-resistant *Enterococcus* spp
ECF: *Enterococcus faecalis*
BHI: Brain–Heart Infusion
LB: Luria–Bertani broth
HD5: Human α-defensin-5
IPTG: Isopropyl-β-d-thiogalactoside
SUMO: Small ubiquitin-like modifier
Trx: thioredoxin
GST: Glutathione *S*-transferase
BCCP: Biotin carboxyl carrier protein
GFP: Green fluorescent protein
