# Supplementary material for: A new generation of recombinant polypeptides combines multiple protein domains for effective antimicrobial activity
Source: Microb Cell Fact. 2020 Jun 5;19:122. doi: 10.1186/s12934-020-01380-7 (PMC7275485; doi:10.1186/s12934-020-01380-7)
Supplement: Supplementary file 1 — Additional file 1: Figure S1. Aminoacidic sequence of JAMF1 for each of the construct domains. Figure S2. Western Blots of JAMF1. (a) Expression time course of JAMF1 at 0, 1, 3 and 5 h post-induction with IPTG in the insoluble and soluble fractions. MWM = Molecular Weight Marker (kDa), IF = Insoluble fraction, SF = Soluble fraction. (b) Western blot of the purified JAMF1 by IMAC (PP) and the purified IBs (PIBs). [file 12934_2020_1380_MOESM1_ESM.docx]

Additional information

**A new generation of recombinant polypeptides combines multiple protein domain for effective antimicrobial activity**

Ramon Roca-Pinilla, Adrià López-Cano, Cristina Saubi, Elena Garcia-Fruitós* and Anna Arís*

Department of Ruminant Production, Institute of Agriculture and Food Research (IRTA), 08140 Caldes de Montbui, Spain

*Corresponding authors. Tel: + 34 93 467 40 40; Fax: +34 93 467 40 42; E-mail: anna.aris@irta.cat, [elena.garcia@irta.cat](mailto:elena.garcia@irta.cat)


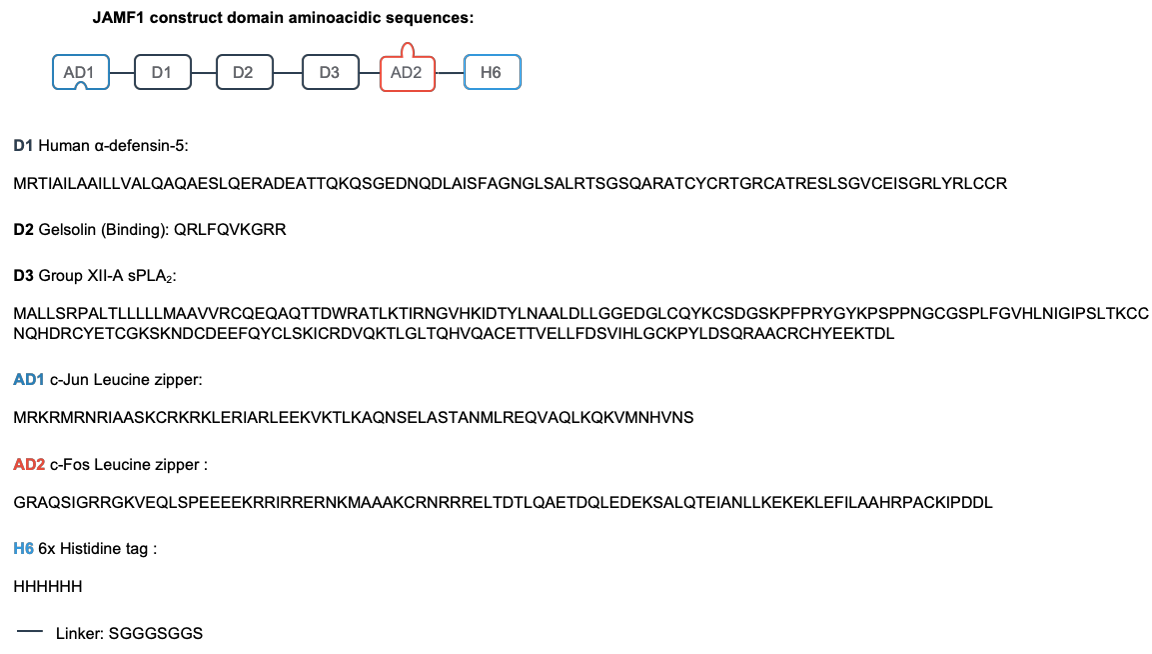


Figure S1. Aminoacidic sequence of JAMF1 for each of the construct domains.


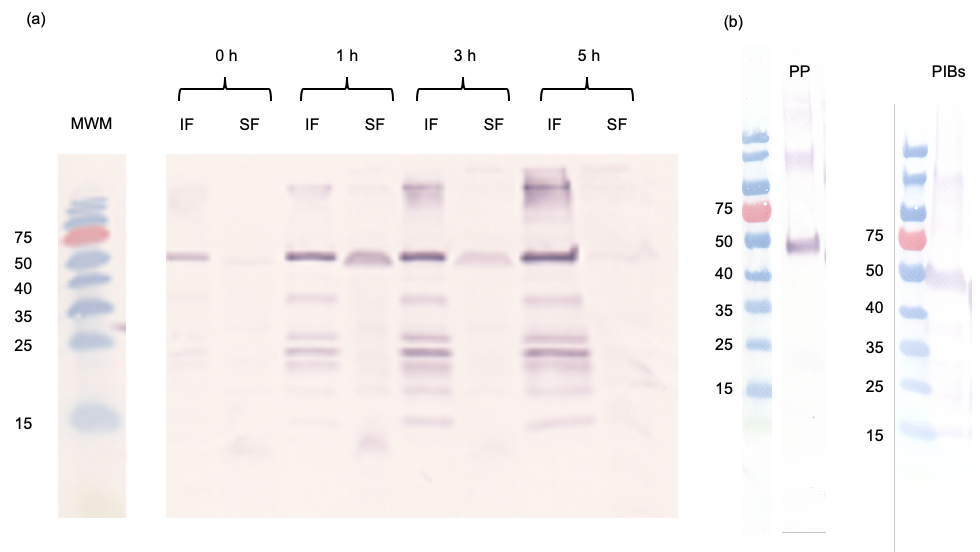


Figure S2. Western Blots of JAMF1. (a) Expression time course of JAMF1 at 0, 1, 3 and 5 h post-induction with IPTG in the insoluble and soluble fractions. MWM = Molecular Weight Marker (KDa), IF = Insoluble fraction, SF = Soluble fraction. (b) Western blot of the purified JAMF1 by IMAC (PP) and the purified IBs (PIBs).
